# Antisense transcription regulates the expression of sense gene via alternative polyadenylation

**DOI:** 10.1007/s13238-017-0497-0

**Published:** 2017-12-22

**Authors:** Ting Shen, Huan Li, Yifan Song, Jun Yao, Miao Han, Ming Yu, Gang Wei, Ting Ni

**Affiliations:** 1 Ministry of Education (MOE) Key Laboratory of Contemporary Anthropology, Collaborative Innovation Center of Genetics and Development, School of Life Sciences and Shanghai Cancer Center, Fudan University, Shanghai, 200438 China; 2 Collaborative Innovation Center of Genetics and Development, Sheng Yushou Center of Cell Biology and Immunology, School of Life Sciences and Biotechnology, Shanghai Jiao Tong University, Shanghai, 200240 China; 3 State Key Laboratory of Genetic Engineering, Collaborative Innovation Center of Genetics and Development, School of Life Sciences and Huashan Hospital, Fudan University, Shanghai, 200438 China

**Keywords:** natural antisense transcripts, alternative polyadenyaltion, 3′UTR, *RNASEH2C*, *KAT5*

## Abstract

Natural antisense transcripts (NAT) and alternative polyadenylation (APA) of messenger RNA (mRNA) are important contributors of transcriptome complexity, each playing a critical role in multiple biological processes. However, whether they have crosstalk and function collaboratively is unclear. We discovered that APA enriched in human sense-antisense (S-AS) gene pairs, and finally focused on *RNASEH2C*-*KAT5* S-AS pair for further study. *In cis* but not *in trans* over-expression of the antisense *KAT5* gene promoted the usage of distal polyA (pA) site in sense gene *RNASEH2C*, which generated longer 3′ untranslated region (3′UTR) and produced less protein, accompanying with slowed cell growth. Mechanistically, elevated Pol II occupancy coupled with SRSF3 could explain the higher usage of distal pA site. Finally, NAT-mediated downregulation of sense gene’s protein level in *RNASEH2C*-*KAT5* pair was specific for human rather than mouse, which lacks the distal pA site of *RNASEH2C*. We provided the first evidence to support that certain gene affected phenotype may not by the protein of its own, but by affecting the expression of its overlapped gene through APA, implying an unexpected view for understanding the link between genotype and phenotype.

**Electronic supplementary material:**

The online version of this article (10.1007/s13238-017-0497-0) contains supplementary material, which is available to authorized users.

## Introduction

Eukaryotic transcriptome has exhibited increasing complexity due to the discovery of gene regulation at multiple aspects (Licatalosi and Darnell, 2010), such as dynamic variation in transcription initiation, alternative splicing, alternative 3′ end processing and RNA localization, etc. There exist many gene loci characterized with sense and overlapping natural antisense transcripts (NATs) in many species (Katayama et al., 2005; Yelin et al., 2003; David et al., 2006). *Cis*-encoded NATs are driven by promoters at the opposite strand of the so-called sense gene, usually partially reverse complementary to its sense partner. The sense-antisense (S-AS) pairs were found to express synergistically rather than by chance (Chen et al., 2005). In S-AS gene pairs, sense gene usually refers to the protein-coding gene, while the antisense partner can be either coding or non-coding. NATs can affect the expression of corresponding sense genes *in cis* or *in trans* at multiple levels (Pelechano and Steinmetz, 2013). One possible mechanism is that NATs can act as a scaffolder to recruit *trans*-factors to the sense gene loci, and affect its transcription by changing locally the state of DNA methylation or histone modification (Pelechano and Steinmetz, 2013). Splicing processes can be modulated by NATs as well (Beltran et al., 2008; Hu et al., 2016). A recent study reported that an antisense transcript, 5S-OT, modulated alternative splicing *in trans* through Alu or anti-Alu pairing with target gene (Hu et al., 2016). NATs can also influence the stability and translation of transcripts via the formation of sense-antisense double-stranded RNA (dsRNA) (Faghihi et al., 2010; Carrieri, 2012).

Alternative polyadenylation (APA), defined as the polyadenylation of precursor messenger RNA (pre-mRNA) at multiple sites, is another layer of gene regulation that contributes to transcriptome complexity at the last step of mRNA maturation (Elkon et al., 2013; Colgan and Manley, 1997). APA has been demonstrated to play critical roles in biological and pathological processes such as development, tissue identity, cell proliferation, cell differentiation, as well as cancer and heart failure (Ji et al., 2009; Ni et al., 2013; Sandberg et al., 2008; Ji and Tian, 2009; Mayr and Bartel, 2009; Fu et al., 2011; Park et al., 2011). Most often, APA generates transcript variants with different length of 3′ untranslated regions (3′UTR), though it could affect the coding region occasionally. Different length of 3′UTR could affect RNA stability and translation efficiency mediated by either microRNA (miRNA) or RNA binding protein (RBP) (Sandberg et al., 2008; Mayr and Bartel, 2009). Besides, different 3′UTR can also affect the subcellular localization of RNAs or corresponding proteins (An et al., 2008; Berkovits, 2015). So far as is known, APA can be regulated by *cis*-acting elements, *trans*-acting factors, Pol II occupancy and elongation rate, and chromatin state (Di Giammartino et al., 2011; Millevoi and Vagner, 2010; Ji, 2011; Pinto et al., 2011; Gunderson et al., 1998; Kaida et al., 2010; Spies et al., 2009).

There are more than 30% annotated human transcripts containing NATs (Ozsolak et al., 2010), and around 70%–75% human genes have APA (Elkon et al., 2013; Derti et al., 2012). Based on such prevalence of antisense transcription and APA in human genome, we speculate that they may crosstalk and function collaboratively in certain cases. A known example is that expression of NATs associates with the relative abundance of two sense isoforms generated by APA in mouse embryonic stem cells (Onodera et al., 2012). However, whether and how NATs regulate APA is completely unknown. Discovery of the interaction of these events will broaden our understanding of transcriptome complexity and form additional connections from genotype to phenotype.

To dive into this question, we first analyzed our published PA-seq data generated from 13 human tissues and found that APA had a significant enrichment in sense-antisense gene pairs, among of which the S-AS gene pair *RNASEH2C*-*KAT5* was selected to address the causality between antisense transcription and APA. We found that *in cis* but not *in trans* over-expression of antisense *KAT5* promoted the higher usage of distal pA site of sense *RNASEH2C* gene. Unexpectedly, *in cis* increased expression of *KAT5* led to a dramatic protein decline of *RNASEH2C,* which successively led to decreased cell proliferation rate. Pol II occupancy and recruited SRSF3 were found associated with higher usage of distal pA site, and noteworthy, such regulation for *RNASEH2C*-*KAT5* existed in human but not in mouse, suggesting this is a newly evolved mechanism and adds a hidden layer of transcriptome diversity in human genome. Together, we discovered for the first time that antisense transcription regulated sense gene’s expression through alternative polyadenylation.

## Results

### APA enriched in overlapped gene pairs

To explore whether antisense transcripts and APA have possible connections genome-widely, we analyzed our previously published PA-seq datasets from 13 human tissues (Ni et al., 2013), and found that sense-antisense (S-AS) genes accounted for 23.33% of the expressed genes (3,471/14,876), similar to the proportion previously reported (Ozsolak et al., 2010). Interestingly, genes with S-AS pairs had more numbers of APA gene than the rest genes (Table S1, 1.24-fold enrichment, *X* ^2^ test, *P* value = 1.38 × 10^−10^). Then, tail-to-tail S-AS gene pairs were chosen for further study since they overlapped in the polyadenylation sites and more likely to have mechanistic interaction between antisense transcription and APA. Compared to non-overlapped genes, tail-to-tail S-AS gene pairs were found more enriched with APA genes (Table S1, 1.19-fold enrichment, *X* ^2^ test, *P* value = 2.95 × 10^−16^), implying intrinsic relevance between antisense transcription and APA.

Since distal polyA (pA) site of one gene in tail-to-tail S-AS gene pair always stayed on the way of the transcription of the other gene, we next examined the correlation between change of NATs expression and the distal pA site usage of the sense gene by PA-seq, which can quantify both the distal/proximal pA site usage and the relative gene expression level (Ni et al., 2013). Interestingly, we found both positive and negative correlations (Fig. S1), suggesting the link between NATs expression and distal pA site usage was rather complicated. To probe into whether antisense transcription played a causal role in regulating pA site usage of sense gene, we applied candidate gene approach following the criteria: 1) relative high expression of both genes in a S-AS gene pair in at least 10 out of 13 human tissues; 2) distal and proximal pA sites are both used in all 13 tissues; 3) relatively high correlation coefficient between antisense transcription expression and distal pA site usage; 4) novel pA site detected by PA-seq (not annotated by RefSeq), which would likely indicate new functional aspects of known gene. Finally, *RNASEH2C*-*KAT5* S-AS gene pair was selected for extensive investigation, because this pair met all the criteria above, and both genes in the pair were protein-coding and has molecular function related to genome stability and DNA repair, which have been reported involved in important biological processes such as cancer and aging (Loeb, 2011; Wallace et al., 2012; Lopez-Otin et al., 2013; Moskalev et al., 2013). *RNASEH2C* encodes a catalytic subunit of RNASEH2, which supervises genome integrity and stability during DNA replication (Reijns et al., 2012). *KAT5* is a lysine acetyltransferase and plays roles in DNA repair and apoptosis through histone acetylation (Ikura et al., 2000; Squatrito et al., 2006). Interestingly, *RNASEH2C* has two pA sites while *KAT5* has only one pA site, we thus defined *RNASEH2C* as the sense gene and *KAT5* as its antisense partner, and utilized them to examine the effect of antisense transcription on sense gene’s APA.

### Transcript of *RNASEH2C* using the distal pA site is less stable and produces less protein

**Figure 1:**
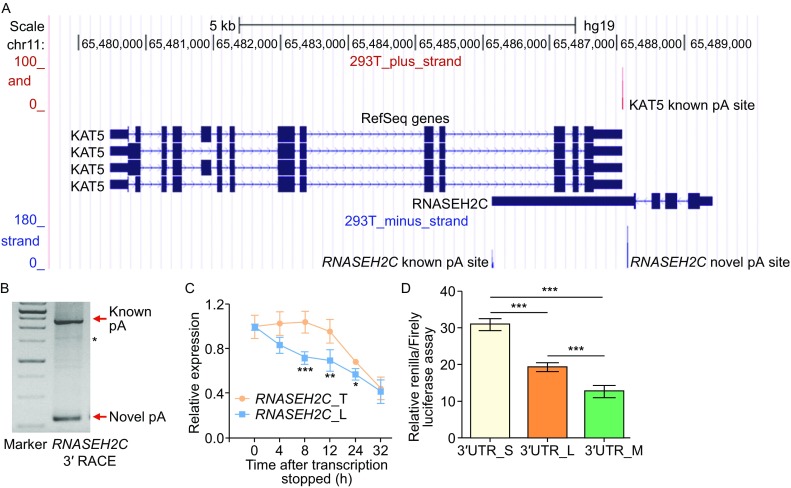
**Transcripts of human** ***RNASEH2C*** **gene with shorter 3′UTR generated by a novel pA site is more stable and produces more protein**. (A) UCSC genome browser track of pA site signal for *RNASEH2C* and *KAT5* based on PA-seq data in human 293T cells. The red vertical line above denotes pA site signal for *KAT5* gene, and the blue lines below denotes that for *RNASE2HC* gene. (B) 3′ RACE result for *RNASEH2C*. The bands indicated by red arrows were products from the short (novel pA site) and long (known pA site) isoforms, respectively. ‘*’ indicated the non-specific amplification. (C) RNA stability assay using qRT-PCR for the total *RNASEH2C* transcripts (*RNASEH2C*_T) and the isoform with long 3′UTR (*RNASEH2C*_L) in 293T cells after blocking transcription with actinomycin D (ActD). (D) Dual luciferase assay for short (S), long (L) and mutant (M) 3′UTR of *RNASEH2C*. Mutant 3′UTR denoted the long 3′UTR in which the proximal polyadenylation signal ‘AATAAA’ was eliminated by mutating to ‘CAATTG’. ***, ** and * represents *P* value (*t*-test) less than 0.001, 0.01 and 0.05, respectively

Human RefSeq gene annotation showed that *KAT5* and *RNASEH2C* are overlapping pair with single polyadenylation site (Fig. 1A). However, our PA-seq data discovered that *RNASEH2C* has a novel pA site in the 3′UTR (named proximal pA site), resulting in a transcript variant with shorter 3′UTR and not overlaps with *KAT5* (Figs. 1A and S2). Both pA sites of *RNASEH2C* were confirmed by two independent methods. First, public PolyA-Seq track provided by UCSC genome browser confirmed the existence of proximal pA site in multiple human samples (Fig. S3). Second, 3′ RACE (rapid-amplification of 3′ cDNA ends) showed two bands corresponding to distal (or known pA, annotated by RefSeq) and proximal pA sites (Fig. 1B), which was further validated by Sanger sequencing (Fig. S4). To inquire the difference between these two isoforms using these two pA sites, qRT-PCR (quantitative reverse transcription real-time polymerase chain reaction) was performed and demonstrated that transcript using the distal pA site (with longer 3′UTR) was less stable than the shorter one upon transcription blocking (Fig. 1C). Interestingly, dual luciferase assay showed that transcript with longer 3′UTR produced less protein than the shorter one (Fig. 1D). These data suggested that alternative polyadenylation of *RNASEH2C* can affect the protein abundance.

### NAT regulates pA site usage of *RNASEH2C in cis*

**Figure 2:**
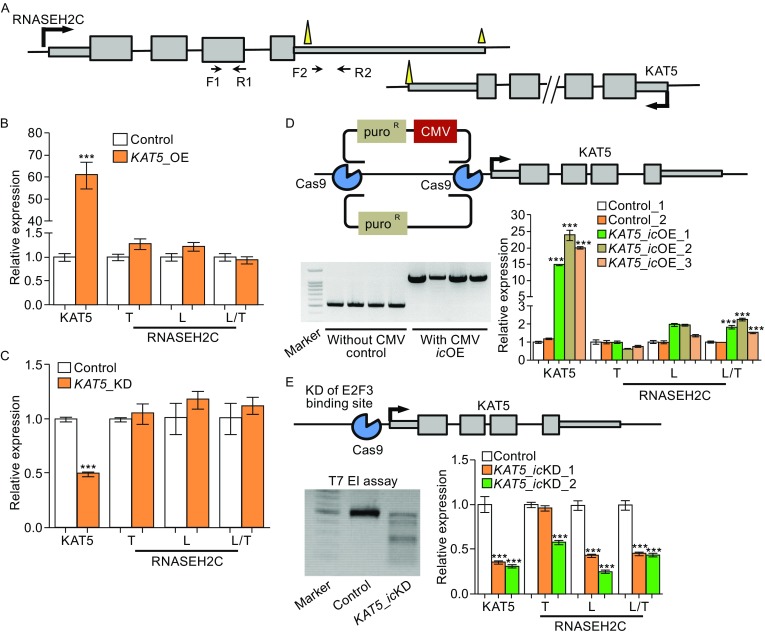
**Antisense transcription** ***in cis*** **regulates alternative polyadenylation of sense** ***RNASEH2C*** **gene**. (A) The schematic diagram denoting the locations of primers used to detect transcripts of *RNASEH2C*. (B and C) Relative expression of *KAT5*, the total (T), long transcript (L) of *RNASEH2C* and the ratio (L/T) of long isoform to the total expression of *RNASEH2C* when over-express (OE, B) or knock-down (KD, C) *KAT5 in trans*. (D) Over-expression of *KAT5 in cis* (*ic*OE) through CRISPR/Cas9 in 293T cells. The upper panel illustrated gene editing strategy on *KAT5*. Arrow denoted the transcription start site (TSS). The gel image at bottom left presented the successful integration of *puro* ^R^ or *puro* ^R^-CMV fragment into the upstream of *KAT5*’s TSS. The bottom right panel presented the relative expression of *KAT5*, *RNASEH2C*_T, *RNASEH2C*_L and relative usage of long 3′UTR (L/T) in *RNASEH2C* upon *KAT5* over-expression *in cis* (*KAT5*_*ic*OE), evaluated by qRT-PCR in three single-cell clones (*ic*OE_1, _2 and _3). (E) Knock-down *KAT5 in cis* (*ic*KD) by CRISPR/Cas9 in 293T cell. The gene editing design was illustrated on the upper panel. The bottom left panel showed the editing efficiency evaluated by T7 endonuclease (EI) assay. The bottom right panel presented the relative expression of *KAT5*, *RNASEH2C*_T, *RNASEH2C*_L and relative usage of long 3′UTR (L/T) in *RNASEH2C* upon *KAT5* knock-down *in cis* evaluated by qRT-PCR in two single-cell clones (*ic*KD_1 and _2). *** represents *P* value (*t*-test) less than 0.001

The difference in translation outcome between isoforms of *RNASEH2C* using different pA sites indicated biological importance of APA dynamic changes in human tissues. Interestingly, the usage of distal pA site of *RNASEH2C* was found positively correlated with *KAT5* expression (Fig. S1), which was further validated in 6 additional human cell lines (Fig. S5). To investigate further the effect of NAT in controlling pA site usage of the sense gene, we perturbed the expression of NAT gene (*KAT5*) and then measured the pA site usage in *RNASEH2C*. Interestingly, we found that ectopic *(*or *in trans)* overexpression of *KAT5* did not affect the pA site usage of *RNASEH2C* (Fig. 2A and 2B), neither did RNA interference (RNAi)-mediated knockdown of *KAT5* (Fig. 2C). Then, we manipulated *KAT5* expression *in cis* by replacing its original promoter with a stronger mammalian CMV promoter through CRISPR/Cas9 gene editing method (Fig. 2D). Intriguingly, unlike *in trans* overexpression of NAT, *in cis* elevation of NAT caused a significant higher usage of distal pA site in *RNASEH2C* by both qRT-PCR and Northern blot (Figs. 2D and S6). Consistently, *in cis* knockdown of *KAT5* by deleting a core binding motif of transcription factor (E2F3) using CRISPR/Cas9 approach led to a significant lower usage of distal pA site of *RNASEH2C* (Fig. 2E). These intervention results were in consistent with the positive correlation between NAT expression and distal pA site usage in multiple human tissues and cell lines (Figs. S1 and S5). Thus, *in cis* transcription of NAT, rather than *in trans* expression, regulated pA site usage of the sense gene in *RNASEH2C*-*KAT5* S-AS gene pair.

**Figure 3:**
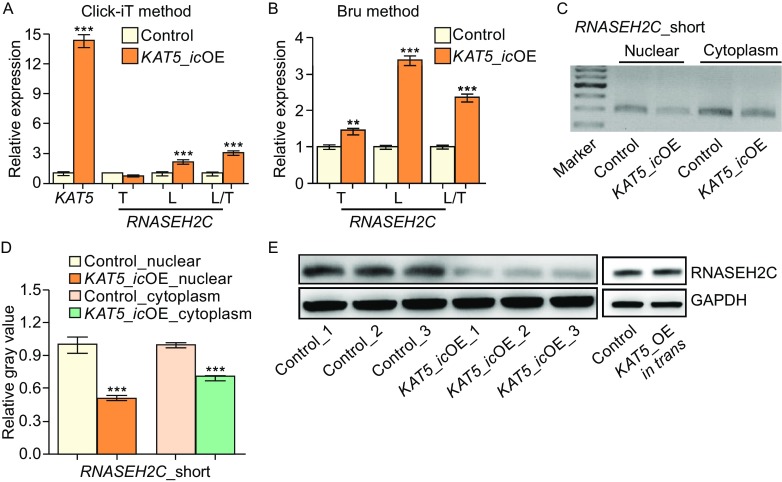
***In cis*** **elevated antisense transcription leads to higher usage of distal pA site in nascent RNA and decreased protein production**. (A) Relative expression of *KAT5*, and different isoforms of *RNASEH2C* in the nascent RNA detected by the Click-iT method. (B) Relative usage of distal pA site of *RNASEH2C* detected by bromouride (Bru) labeling followed by qRT-PCR. (C) Abundance detection of short isoform in nuclear and cytoplasm by RT-PCR and electrophoresis. (D) The short isoform abundance quantification using the relative gray value for band signal in C by imageJ. (E) Western blot for human RNASEH2C upon *in cis* and *in trans* over-expression of *KAT5*. GAPDH served as the intern control. All the results showed in Fig. 3 were from single-clone cells

Since steady-state mRNA level is determined by the rates of nascent RNA transcription and RNA degradation, to precisely examine whether antisense transcription regulated APA at transcriptional level, abundance of nascent RNA was evaluated by two independent approaches (Click-iT and Bru-PCR) (Jao and Salic, 2008; Paulsen et al., 2014). *In cis* transcriptional upregulation of *KAT5* was first confirmed (Fig. 3A). Next, higher usage of distal pA site for *RNASEH2C* at nascent RNA level was validated by both Click-iT and Bru-PCR methods (Figs. 3A, 3B and S7). These evidences suggested that NAT controlled APA of the sense gene at transcriptional level.

### *In cis* upregulated antisense transcription leads to less protein production of the sense gene

To explore the consequence of APA, we quantify the abundance of *RNASEH2C* isoform with short 3′UTR in cells with *in cis* overexpression of *KAT5*, because it was possibly the major template for translation (Fig. 1A and 1D). Consistent with this expectation, reduced abundance of the short *RNASEH2C* isoforms was detected in cytoplasmic fraction (Figs. 3C, 3D and S8). Accordingly, reduced protein level of RNASEH2C was found in both single clones (Fig. 3E) and mixed cells with *in cis* overexpression of *KAT5* (*KAT5*_*ic*OE) (Fig. S9). In contrast, *in trans* overexpression of *KAT5* did not affect the protein level of RNASEH2C (Fig. 3E). These results above collectively demonstrated that *in cis* overexpression of *KAT5* led to higher distal pA site usage, then led to decreased mature mRNA template for translation in cytoplasm, and finally less protein generation.

### Decreased RNASEH2C protein production slows cell growth

**Figure 4:**
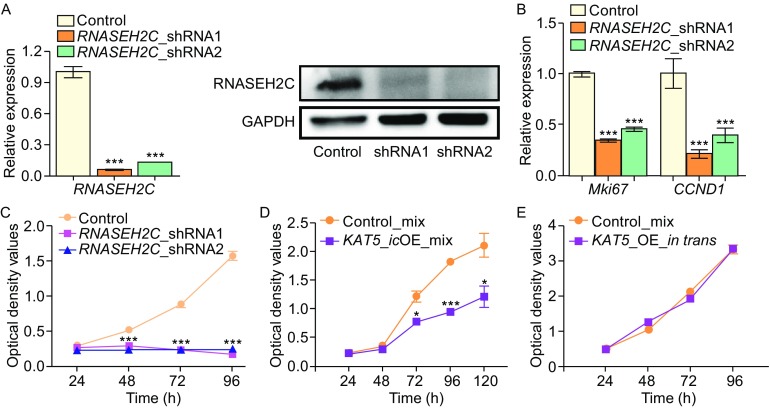
**Reduced RNASEH2C protein abundance leads to slower cell proliferation rate**. (A) qRT-PCR (left) and Western-blot (right) for RNASEH2C after knockdown *RNASEH2C* by two shRNAs. GAPDH served as the internal control. (B) Relative expression level of *Mki67* and *CCND1* evaluated by qRT-PCR. (C) Cell proliferation rate in *RNASEH2C*-knockdown cells compared to control cells assayed by CCK-8 kit. (D) CCK-8 assay for cells *in cis* overexpressing of *KAT5* (*KAT5*_icOE_mix) and control 293T cells. (E) Comparison of cell growth rate between control cells and cells *in trans* over-expressing *KAT5* (*KAT5*_OE_*in trans*) by CCK-8 assay. Overexpression of *KAT5 in trans* was performed using lentivirus transduction system

The cellular and molecular phenotypes of reduced RNASEH2C level were next investigated. Cell growth ability dramatically decreased in *RNASEH2C*-depleted human 293T cells (Fig. 4A), and the expression of related molecular makers, such as *Mki67* (the replication marker) and *CCND1* (encodes Cyclin D1, an important cell cycle marker), were also sharply declined (Fig. 4B and 4C). Additionally, similar phenotypes were also observed in HUVEC (human umbilical vein endothelial cells) and A549 (human lung adenocarcinoma cell line) cells upon knockdown of *RNASEH2C* (Fig. S10).

Since the abundance of RNASEH2C protein significantly reduced upon *in cis* over-expression of *KAT5*, we then examined the cell growth behavior in cells over-expressing *KAT5 in cis*. Interestingly, these cells also exhibited reduced cell growth rate (Fig. 4D), which was similar to *RNASE2HC*-depleted ones. In contrast, ectopic over-expression of *KAT5* did not have detectable effect on cell proliferation (Fig. 4E), which excluded the possibility that phenotypes presented above were resulted directly from the increased protein level of *KAT5*.

### High Pol II occupancy coupled with SRSF3 is associated with distal pA site usage

**Figure 5:**
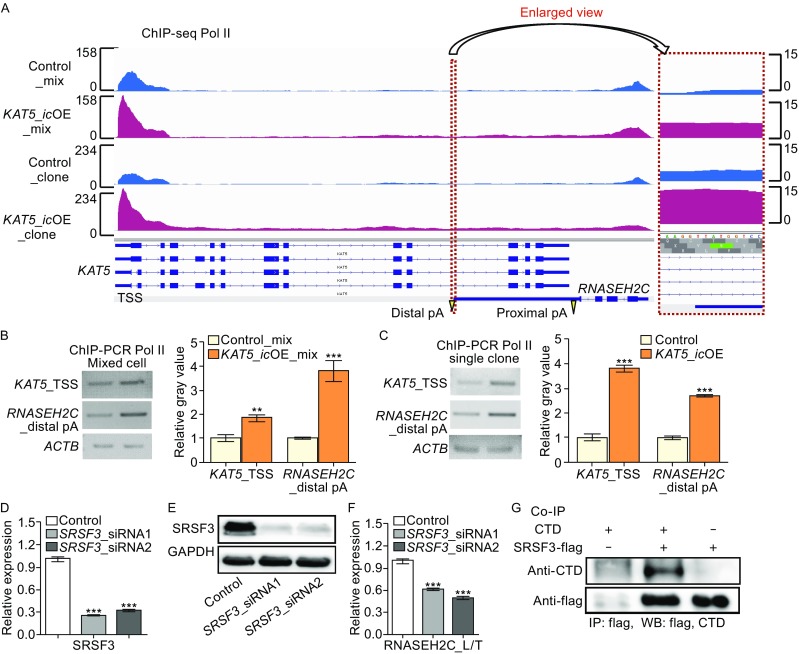
**Higher Pol II occupancy and the recruitment of SRSF3 are associated with increased usage of distal pA site in** ***RNASEH2C***. (A) Pol II profiling on the *RNASEH2C* and *KAT5* genes by ChIP-seq. Po II ChIP-seq was performed on both mixed cells directly after gene editing (postfixed with mix) and single-clone-derived cells (postfixed with clone). The region nearby distal pA site of *RNASEH2C* was highlighted by red dashed rectangles and the enlarged view was presented in the right panel. (B and C) ChIP-PCR validation at *KAT5* promoter region and distal pA site of *RNASEH2C* on mixed cells (B) and cells derived from single clone (C). Relative gray value quantified by imageJ was shown on the right. Beta-actin gene (*ACTB*) served as control. (D and E) Knockdown of SRSF3 by two siRNAs in 293T cells confirmed by qRT-PCR (D) and Western blot (E). (F) The relative expression of long isoform to total (L/T) of *RNASEH2C* in SRSF3-depleted cells and the control, quantified by qRT-PCR. (G) Co-IP detection for the interaction of SRSF3 and the CTD of Pol II. *, ** and *** represents *P* value (*t*-test) less than 0.05, 0.01 and 0.001, respectively

As both *RNASEH2C* and *KAT5* in this S-AS gene pair are protein-coding genes and transcribed by RNA polymerase II (Pol II), which has been demonstrated to play regulatory roles in alternative polyadenylation (Ji, 2011; Pinto et al., 2011; Hsin and Manley, 2012), we thus performed Chromatin immunoprecipitation followed by deep sequencing (ChIP-seq) for Pol II to probe into the possible mechanism by which antisense transcription regulated APA. ChIP-seq result showed higher Pol II occupancy near promoter region of *KAT5* in both human cell mixture and cells derived from single clones of CRISPR/Cas9 gene editing (*KAT5*_*icOE*) compared to control cells (Fig. 5A). Such results were further confirmed by ChIP-PCR and ChIP-qPCR (Figs. 5B, 5C and S11). Intriguingly, higher Pol II occupancy was detected at both distal and proximal pA sites of sense gene *RNASEH2C* upon *in cis* overexpressing *KAT5* (Figs. 5A–C and S11).

To explain the higher usage of distal pA site upon elevation of Pol II occupancy, we hypothesize that distal pA site is more sensitive to local Pol II concentration compared to the proximal one. Two lines of evidence support this hypothesis. First, distal pA site has much lower usage than proximal one in most of the human tissues (Figs. 1A, S2 and S3), implying *cis*-regulatory strength is relatively weak near distal site. Second, distal pA site is newly evolved (Fig. S12), thus the *cis*-regulatory elements of polyadenylation might be not as strong as the conserved proximal one, and the usage of distal pA site possibly needs more *trans*-acting factors recruited by Pol II to help.

To further investigate if existed proteins recruited by Pol II can regulate the selection of distal pA site in *RNASEH2C*, we screened 14 genes encoding 3′ end processing factors or splicing factors in 293T cells. Interestingly, splicing factor SRSF3 showed the most significant impact on changes in pA site usage (Figs. 5D–F and S13). In the test cells with SRSF3 knockdown, decreased ratio of the long transcript was observed, implying that usage of the distal pA site of *RNASEH2C* was inhibited upon SRSF3 knockdown. Moreover, further study provided evidence that SRSF3 interacted with the C terminal domain of Pol II (Fig. 5G), which is consistent with previous findings (de la Mata and Kornblihtt, 2006). Collectively, we speculated that elevated Pol II coupled with SRSF3 might participate in the regulation of *RNASEH2*’s APA.

### NAT-mediated APA regulation in *KAT5*-*RNASEH2C* gene pair is a newly evolved mechanism in controlling protein production of sense gene

**Figure 6:**
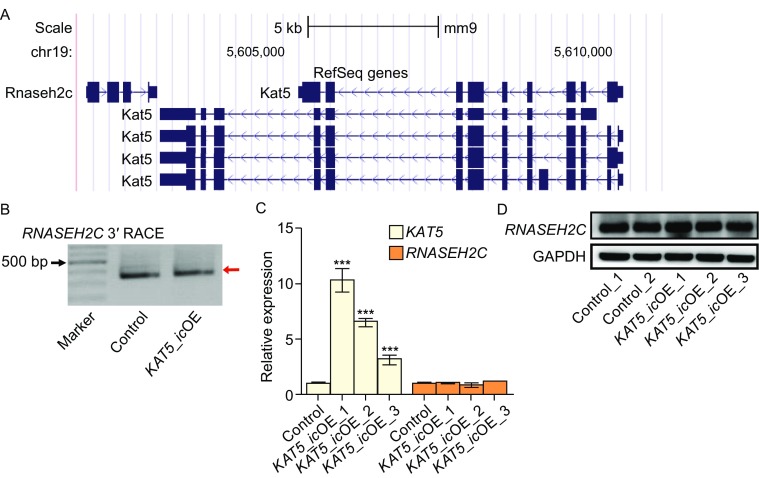
**Overlapping pA site is required for antisense transcription mediated downregulation of protein production**. (A) Gene structures of *RNASEH2C* and *KAT5* based on mouse RefSeq gene annotation in UCSC genome browser. (B) 3′ RACE results for *RNASEH2C* in mouse NIH3T3 cells with or without *KAT5 in cis* over-expression (*KAT5_ic*OE). (C) Relative expression of *KAT5* and *RNASEH2C* in *KAT5_ic*OE and control mouse cells, quantified by qRT-PCR. (D) Western blot for RNASEH2C in *KAT5_ic*OE and control mouse cells. GAPDH served as the internal control. All the results showed in this figure were from single-clone cells

To ask the biological significance of antisense transcription mediated downregulation of sense gene’s protein production in an evolutionary view, we examined the conservation of DNA sequences near the distal pA site of *RNASEH2C* in multiple species. The results revealed that only primates had distal polyA signal in *RNASEH2C* gene (Fig. S12A), while other mammalians such as mouse and rat did not have the distal polyA signal, resulting the absence of isoform with long 3′UTR (Figs. 6A and S14). Moreover, upstream DNA sequence of distal pA site was less conserved than that of proximal one (Fig. S12B).

Our data suggested that *in cis* overexpression of antisense transcript leading to downregulation of overlapping gene’s protein level was mediated by distal pA site preference in human cells. To demonstrate that distal pA site played a critical role in mediating the reduced protein production, we *in cis* overexpressed *KAT5* in mouse cells, which lacks the distal pA signal in *RNASEH2C* gene. 3′ RACE and Sanger sequencing confirmed that *KAT5*_*ic*OE mouse cells kept to use only one pA site (corresponding to human proximal pA site) (Figs. 6B and S14). Further, unlike that in *KAT5*_*ic*OE human cells, no change in RNA level and protein abundance of *RNASEH2C* was detected in *KAT5*_*ic*OE mouse cells (Fig. 6C and 6D). All these data indicated that, in S-AS gene pairs like *KAT5*-*RNASEH2C*, antisense transcription mediated APA regulation acted as a novel and intriguing mechanism in regulating the expression of the overlapped gene, adding a new layer of complexity in human gene regulation.

## Discussion

**Figure 7:**
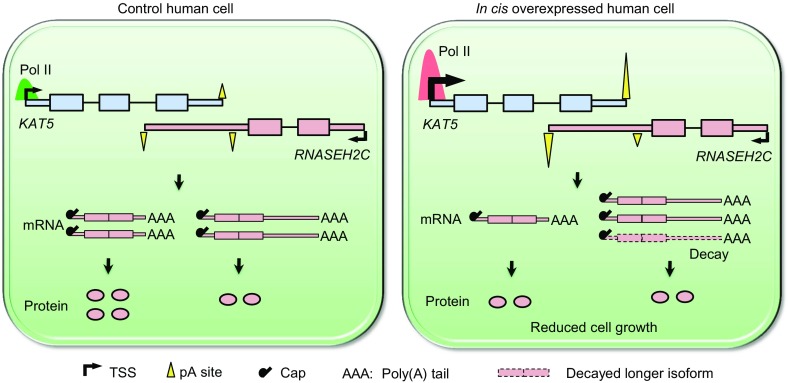
**Working model for antisense transcription mediated regulation on sense gene via APA**. *RNASEH2C* and *KAT5* are tail-to-tail overlapping in human genome. Overexpression of *KAT5 in cis* caused the elevated occupancy of Pol II on *KAT5* and extended to *RNASHE2C* gene body, leading to the preference of distal pA site in *RNASHE2C*. Higher distal pA site usage of *RNASEH2C* resulted in increased abundance of isoform with longer 3′UTR, which generated less protein than the shorter one. In addition, transcripts with longer 3′UTR had higher degradation rate, further aggravated the reduction in RNASEH2C protein production. Thus *in cis* elevated antisense transcription down-regulated sense gene’s protein level, leading to reduced cell growth rate

Understanding the mechanism contributing to the complexity of transcriptome is one of the key issues in post-genomic era, which will help to bridge the gap from genotype to phenotype. Due to the universal existence of S-AS gene pairs genome-wide, the causality of phenotype sometimes need to take both target gene and its neighbors into consideration. As an example, targeting therapy of *ERBB2*, an important regulator of breast cancer, turns out to be not successful because of the co-amplification and over-expression of its neighbor genes (Vanden Bempt et al. 2007; Hu et al., 2009). A second example is the *TNFAIPI*-*POLDIP2* S-AS gene pair, whose co-regulated expression is associated with breast cancer phenotypes and patient survival, suggesting the therapeutic approach needs to take genes in this complex sense-antisense architecture into consideration (Grinchuk et al., 2010). Given such importance of collaborative role in nearby gene pairs, our finding that NAT affected overlapped sense gene’s expression through APA revealed another layer of transcriptional complexity that bridges genotype and phenotype. In the present study, we showed that *in cis* over-expression of *KAT5* led to reduced cell proliferation not through increased protein level of *KAT5* itself, but through transcriptional interference on its overlapped gene *RNASEH2C* via APA (Fig. 7). Such an unexpected mechanism broadens our knowledge in gene expression regulation and opens up a new way to search for key genes controlling physiological/pathological phenotypes.

S-AS gene pair can either be coding or non-coding. In the case of present study, both *RNASEH2C* and *KAT5* are coding genes. We defined *RNASEH2C* as sense gene here since it contained two pA sites, which generated two distinct 3′UTRs that can affect protein production. *KAT5* was then defined as antisense gene accordingly. It should be mentioned that although human *KAT5* had four splicing isoforms, they shared the same transcriptional start site and single polyadenylation site, and did not contain alternative splicing events in the overlapped region (Fig. 1A), thus *in cis* overexpression of *KAT5* won’t introduce additional factors affecting APA of *RNASEH2C*. The experimental results indicated that antisense-mediated regulation of sense gene depended on the overlapped distal pA site. The existence of distal pA site in human while lacks in mouse in *RNASEH2C* provides an excellent comparison pair to test the hypothesis. In human cells, elevated transcription of antisense gene (*KAT5*) caused a distal pA site usage shift in sense gene (*RNASEH2C*), which reduced the production of corresponding protein. However, in mouse cells, due to the absence of distal pA site, *in cis* overexpression of *KAT5* cannot trigger APA, and result in the unchanged expression of *RNASEH2C*.

Pol II has been reported to regulate APA (Ji, 2011; Pinto et al., 2011; Hsin and Manley, 2012). Moreover, high transcriptional activity (usually corresponding to elevated Pol II occupancy) was also found promote proximal pA site usage of the same gene (Ji, 2011). In the case of tail-to-tail *RNASEH2C*-*KAT5* gene pair, distal pA site is far away from *RNASEH2C* promoter, but is much closer to *KAT5* promoter (Fig. 1A). Thus *in cis* overexpression of *KAT5* favored the usage of pA site closer to *KAT5* promoter (i.e., the distal pA site of *RNASEH2C*), consistent with the conclusion drawn by Ji et al. (2011). Previous studies have already showed that RNA processing factors including splicing factors and polyadenylation factors can bind to the C terminal domain (CTD) of Pol II to participate in RNA processing (Hsin and Manley, 2012; Di Giammartino et al., 2011). Intriguingly, we discovered splicing factor SRSF3 could bind to the C terminal domain of Pol II to contribute to pA site selection of *RNASEH2C*. However, it is worth noting that such a mechanism might not be general in all tail-to-tail gene pairs given the correlation coefficient between antisense expression and distal pA site usage can be either positive or negative (Fig. S1).

Over-expressing *KAT5 in cis* did not downregulate RNASEH2C protein level in mouse cells, suggesting the newly evolved distal pA site in human was critical to mediate APA in *RNASEH2C* and ultimately reduced the protein level. We speculated that such function of pA site usage might depend on the interplay between the *cis*-regulatory elements and *trans*-acting factors. Although the ideal experimental design to prove the importance of APA in down-regulating sense gene’s protein level was to completely disrupt the usage of distal pA site in *RNASEH2C*, it was technical challenging to delete all the *cis*-elements near the distal pA site completely. Actually, we tried multiple gRNAs and still can not obtain cell clones with mutated polyA signal upstream of the distal pA site, possibly due to the inefficiency of the related gRNA (Fig. S15A). Nevertheless, we successfully deleted polyadenylation-related *cis*-elements downstream of the distal pA site and observed the decreased expression of the long isoform of *RNASEH2C* (Fig. S15B and S15C), suggesting that these elements were helpful for the recognition and cleavage of the distal pA site. To circumvent this technical limitation, we introduce mouse cells that lack of distal pA site for RNASEH2C to serve as an indirect evidence instead, wherein, *in cis* upregulation of *KAT5* could not affect the expression of *RNASEH2C* (Fig. 6). These above results indicated that APA probably played a regulatory role in mediating *RNASEH2C* expression. Although the detailed mechanism of this intriguing regulation awaited to be elucidated, our results suggested that such a regulation should be taken into consideration in understanding the complicated transcriptome, especially in highly-evolved organism such as human.

## Materials and Methods

### Cell culture, transfection and selection of stable cell lines

All cell lines (293T, HUVEC, A549, NIH3T3) used in this study were cultured in DMEM medium supplemented with 10% FBS at 37°C with 5% CO_2_. For transient transfection, cells were seeded in 6-well plate for 60%–70% confluence one day in advance, then transfected with Lipofectamine 2000 (Invitrogen) on the second day. Cells were harvested for RNA and protein extraction 48 h after transfection.

We adopted CRISPR/Cas9 system for gene editing. For *KAT5* knockdown *in cis*, cells were transfected with 2 μg plasmids (pBC2-gRNA). 24 h after transfection, cells were screened by 300 μg/mL hygromycin for two days, then the survival cells were trypsinized, diluted and seeded into a 96-well plate for single cell clone selection. For *KAT5* overexpression *in cis*, cells were co-transfected with 2 μg pBC2-gRNA plasmid and *puro* ^R^-CMV-T-easy vector, with pBC2-gRNA plasmid and *puro* ^R^-T-easy vector serving as control, respectively. 24 h after transfection, cells were screened by 300 μg/mL hygromycin for two days, and the survival cells were cultured continuously in medium with 2 μg/mL puromycin for ~5 days, during which period, medium was replaced every day to remove the dead cells. The final survival cells were cultured for an additional 7–10 days for single cell clone selection. For distal pA site mutation in human *RNASEH2C*, we adopted an episomal vector-based CRISPR/Cas9 system for higher gene knockout efficiency, and screened for stable single cells according to the published methods (Xie et al., 2017) . Then single-cell-derived clones were selected for further experiment.

For knockdown *RNASEH2C* by lentivirus transduction, shRNA oligonucleotides for *RNASEH2C* were annealed and then cloned into the pLOK.1 vector. Then this vector or control shRNA plus VSVG and gag/pol encoding plasmids was transfected into the 293T cell line, respectively. After cultured for 24 h and 48 h, the virus supernatant was harvested to infect 293T cell, respectively. 24 h post the second infection, the medium was replaced with fresh DMEM medium supplemented with puromycin (2 μg/mL). After one day selection, the survival cells were trypsinized and split into two parts, one for cell proliferation assay, the other part was cultured for additional two days in the medium with puromycin (2 μg/mL). Then the cells were harvested for RNA and protein extraction to validate *RNASEH2C* knockdown efficiency.

### Vector construction

For *RNASEH2C* shRNA plasmid construction, the DNA oligonucleotides were annealed and then cloned into the pLKO.1 plasmid by *Eco*RI and *Age*I restriction site. Sequences corresponding to short or long 3′UTR of *RNASEH2C* were PCR amplified by Q5 high-fidelity DNA polymerase (NEB), and then inserted into the Renilla luciferase vector (psiCHECK-2 vector). Meanwhile, a mutated long 3′UTR was constructed by replacing the proximal polyadenylation signal AATAAA with CAATTG. As to the vector for *in trans* overexpression of *KAT5*, the complete coding sequences (CDS) were acquired by PCR amplification from human 293T cDNA, then the fragments were inserted into the pcDH_EF1_MCS_T2A_Puro plasmid by *Eco*RI and *Bam*HI restriction site.

The vectors for knock-out and knock-in of *KAT5 in cis* were constructed following the description below. For gRNA vectors construction, gRNAs were designed using online CRISPR Design (http://crispr.mit.edu/), and the synthesized oligos were annealed with sticky terminus of *Hpa*I and *Xba*I restriction site, and then integrated downstream U6 promoter in a middle vector PUG3 (U6-gRNA). The U6-gRNA fragments were then inserted into the final plasmid PBC2 by *Sal*I and *AfI*I restriction site. When two or more gRNAs used, one U6-gRNA was first PCR amplified from gRNA-PUG3 by primers with *Xba*I and *Xho*I restriction site and cloned into downstream of another gRNA-PUG3 vector, finally, the tandem U6-gRNA fragments were inserted into the final plasmid PBC2.

For knock-in CRISPR/Cas9, two donor plasmids were constructed based on the skeleton of T-easy vector. One was the control plasmid with insertion of fragments containing anti-*puro* (*puro* ^R^) gene and homologous arms (HA) upstream and downstream *puro* ^R^. The other plasmid is HA-*puro* ^R^-CMV-HA-T-easy vector, among of which, HA sequences were acquired by PCR amplification from 293T genomic DNA, CMV and *puro* ^R^ gene were PCR amplified from the templates of pCDNA3.1 and PT2B vector, respectively. Then they were ligated successively by overlap-PCR and finally inserted into T-easy vector. Q5 high-fidelity DNA polymerase was used for all PCR reactions to minimize the introduction of possible error bases, and Sanger sequencing was applied for fidelity confirmation.

### RNA extraction, cDNA synthesis, qRT-PCR and Western blot

Total RNA and protein was extracted with TRIzol reagent (Sigma) according to the manufacturer’s instruction. For cDNA synthesis, 1 μg total RNA was reverse-transcribed into the cDNA by FastQuant RT Kit (TIANGEN) and oligo (dT) primer. Then quantitative PCR was performed using 2× SYBR mix (KAPA) on Bio-Rad CFX manager machine.

For Western blot, the primary antibody for detecting RNASEH2C (Abcam, cat.no.ab89726), SRSF3 (Abcam, Cat.no.ab198291), C-terminal domain (CTD) of Pol II (CST, Cat.no.2629) and GAPDH (CST, cat.no.2118S) was 1:1000 diluted and incubated for 2 h at room temperature. The second antibody (HRP conjugate, YEASON) was 1:5,000 diluted and incubated for 1 h at room temperature.

### Northern blot

We performed Northern blot to detect the short and long isoforms of *RNASEH2C* according to the manufacturer’s handbook with some modifications (Roche, Cat. No.12039672910). Briefly, denatured RNA was resolved by 1% agarose gel, and then was transfered to NC membrane (BIO-RAD, Cat.no.1620196) overnight at 4°C. Following UV cross-link, the membrane was pre-hybridized in hybridization solutions at 65°C incubator for 2 h and then incubated with Dig-labelled-probe for hybridization overnight. Then the membrane was washed and blocked in blocking solutions for 30 min at room temperature. Membrane was next incubated with antibody solutions (anti-Dig-AP antibody in Blocking solutions) for 30 min and was washed twice for 15 min by wash solutions. Finally, immunological signal was detected after the membrane was equilibrated in detection buffer.

### Co-immunoprecipitation (Co-IP)

The interaction of SRSF3 with the C-terminal domain (CTD) of Pol II was detected by Co-IP experiment. Firstly, 10 μg of vectors (SRSF3-flag_pcDH, CTD_pcDH, SRSF3-flag_pcDH plus CTD_pcDH) was individually transfected into 293T cells by Lipofectamine 2000. Cells were harvested in lysis buffer 24 h post transfection. We removed 20 μL cell lysate as input and the rest was incubated with anti-flag agarose beads (Sigma, Cat.no.A2220) at 4°C for 6 h. The agarose beads mixture was then washed twice according to the vendor’s handbook. Western blot loading buffer was next added to the beads and incubated at 95°C for 10 min to denature protein. Finally, Western blot was performed to detect SRSF3-flag and the CTD of Pol II by anti-flag (Sigma, Cat.no.F1804) and anti-CTD antibody, respectively.

### RNA stability assay

The stability for short and long isoforms of *RNASEH2C* was measured by adding actinomycin D at a final concentration of 5 μg/mL into 293T cells to block transcription. Cells were harvested at the time point of 0 h, 4 h, 8 h, 12 h, 24 h, 32 h, respectively, and total RNA was extracted with the methods described above. The difference in stability was then evaluated by qRT-PCR. The long isoform *RNASEH2C*_L was quantified using primer specific to long 3′UTR, while the overall expression of *RNASEH2C* was quantified using primers shared by both short and long 3′UTRs (*RNASEH2C*_S).

### 3′ RACE

Firstly, total RNA was treated with DNase I to eliminate DNA contamination. Then 500 ng DNA-free RNA was reverse-transcribed into cDNA with SMARTScribe reverse trascriptase (100 U, Clontech). The primer used for reverse transcription is composed of 19 known nucleotides plus a downstream 30 dTs and two-anchoring nucleotides (VN, V = A, G and C, N = A, T, G and C) from 5′ to 3′ end (primer sequences, AAGCAGTGGTATCAACGCAGA GTACTTTTTTTTTTTTTTTTTTTTTTTTTTTTTTVN). The cDNA product was diluted, and PCR was performed with one primer targeting on the gene and the other primer partially same as the primer mentioned above (primer sequences, AAGCAGTGGTATCAACGCAGAGT). Then PCR products were monitored by agarose gel, and validated by Sanger sequencing.

### Dual-luciferase report assay

293T cells were transfected with psiCHECK-2 vectors containing short-3′UTR, long-3′UTR and mutated-3′UTR, respectively, using Lipofectamine 2000 in 24-well plate. After transfection for 24 h, the firefly and renilla luciferase activities were measured one by one according to the manufacture’s instruction (Promega). The final renilla luciferase activity was normalized to the firefly luciferase signal.

### Cell proliferation assay

After selection by puromycin, the survival cells were trypsinized and diluted, then subcultured in 96-well plate, with 2000 cells per well and three replicates for each time point. CCK-8 reagent (*DOJ*INDO) was then added according to the manufacturer’s protocol to each well every 24 h post seeding, lasting for four days. After each treatment, cells were incubated for two hours at 37°C, then the optical density (OD) at 450 nm and 600 nm were measured respectively for each well by microplate reader (Tecan i-control).

### Isolation of nuclear and cytoplasmic fractions

To separate the nuclear and cytoplasmic components, cellular fractionation was performed following the description below. Cells were trypsinized, washed twice with ice-cold 1× PBS, and resuspended in ice-cold lysis buffer CFB (20 mmol/L Tris-HCl (pH 7.4), 150 mmol/L KCl, 100 mmol/L NaF, 1 mmol/L DTT, 1% NP40, 1.5 mmol/L MgCl_2_, RNasin inhibitor (100 U/mL), 1× protease inhibitor). Cell lysate was incubated on ice for 10 min and then centrifuged at 500 ×*g* for 5 min, the supernatant was carefully pipetted into a new tube. Then wash the pellet once with ice-cold lysis buffer, and centrifuge again at 500 ×*g* for 5 min. The supernatants harvested from these two steps were combined to make the cytoplasmic fraction, and the pellet left was the nuclear fraction. Finally, RNA was prepared from each fraction by using TRIzol reagent.

### Nascent RNA analysis

The nascent RNA assay was performed with two methods, the Click-iT method (Jao and Salic, 2008) and the BrU method (Paulsen et al., 2014). Briefly, for the Click-iT method (Invitrogen), EU, a nucleotide analog, was added into the medium and cells were incubated for 1 h at 37°C. Then total RNA was extracted, and the EU-contained RNA was biotinylated. Then the EU-biotinylated RNA was isoloated from the total RNA by Dynabeads MyOne streptavidin T1 (Invitrogen). For the BrU method, the principle was similar, but BrU replacing EU was introduced into the newly transcribed RNA, then BrU-contained RNA was seperated by anti-BrU antibody (BD Pharmingen). Finally, both EU-RNA and BrU-RNA were quantified by RT-qPCR.

### ChIP-seq and ChIP-PCR

Chromatin immunoprecipitation for RNA polymerase II was conducted according to the following procedure modified from the published method (Yu et al., 2015). Firstly, nucleic fraction was isolated from cell lysate, and the chromatin was fragmented via Bioruptor sonication. Then add the fragmented-chromatin to the anti-Pol II antibody (Santa Cruz, sc-899) which has been pre-incubated with protein G beads (Invitrogen), and then incubate overnight. After washing four times with washing buffer, the isolated DNA was de-crosslinked with overnight incubation at 65°C. Then proteinase K was used to digest protein, followed by DNA purification with ZYMO DNA Clean & Concentrator. The purified DNA was subjected for either library preparation for deep sequencing (ChIP-seq) or PCR and quantitative PCR (ChIP-PCR and ChIP-qPCR). For ChIP-seq library construction, end-repair was first performed for the purified DNA, followed by 3′ end dA-adding reaction and Y-shape adaptor ligation. The final sequencing library was obtained by PCR amplification, and then sequenced via Illumina HiSeq 4000. All primer sequences used for qRT-PCR, ChIP-PCR, ChIP-qPCR and gene editing were listed in Table S2.

### Data analysis

For APA statistics analysis in human genes, Human RefSeq genes were classified into overlapped and non-overlapped gene pairs according to their genomic positions. The overlapped gene pairs were further categorized into three sections, i.e., tail-to-tail, head-to-head and containing. We next used the pA peaks obtained from 13 human tissues in our previous study (Ni et al., 2013) to acquire genes having pA site in overlapped and non-overlapped gene pairs, respectively. Pearson correlation coefficient (R) between antisense expression level and the percentage of distal pA site usage was calculated.

For Pol II ChIP-seq data analysis, the 150 nt raw paired-end (PE) ChIP-seq reads were gained after sequencing on Illumina HiSeq 4000. The sequencing quality was first evaluated using FastQC (http://www.bioinformatics.babraham.ac.uk/projects/fastqc/). Then the 60 nt at the 3′ end of each read was cut off to remove the potential contamination of adapters introduced in library preparation. The clean reads were then aligned to human hg38 genome using BWA (v0.7.10) (Li and Durbin, 2009), and the coverage on genome or selected genomic regions were then calculated using the uniquely mapped reads, the track files was generated using bedtools (v2.21.0) (Quinlan and Hall, 2010), and normalized to sequencing depth, and visualized through IGV (Thorvaldsdottir et al., 2013).

## Electronic supplementary material

Below is the link to the electronic supplementary material.

Supplementary material 1 (DOCX 2446 kb)
