# Supplementary material for: Antisense transcription regulates the expression of sense gene via alternative polyadenylation
Source: Protein Cell. 2017 Dec 22;9(6):540–52. doi: 10.1007/s13238-017-0497-0 (PMC5966356; doi:10.1007/s13238-017-0497-0)
Supplement: Supplementary file 1 — Supplementary material 1 (DOCX 2446 kb) [file 13238_2017_497_MOESM1_ESM.docx]

**Antisense transcription regulates the expression of sense gene via
alternative polyadenylation**

Ting Shen, Huan Li, Yifan Song, Jun Yao, Miao Han, Ming Yu, Gang Wei, Ting Ni

**Supplementary Figures**

**
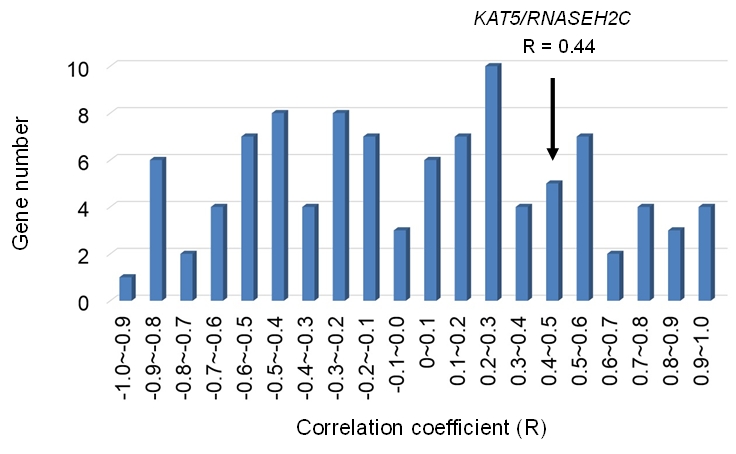
**

**Supplementry Figure S1. The distribution of genes with different correlation coefficient between antisense expression and distal pA site usage in human tissues**. PA-seq data of 13 human tissues were downloaded from the SRA059064 (1). Both positive and negative correlation coefficient values were shown in X axis.


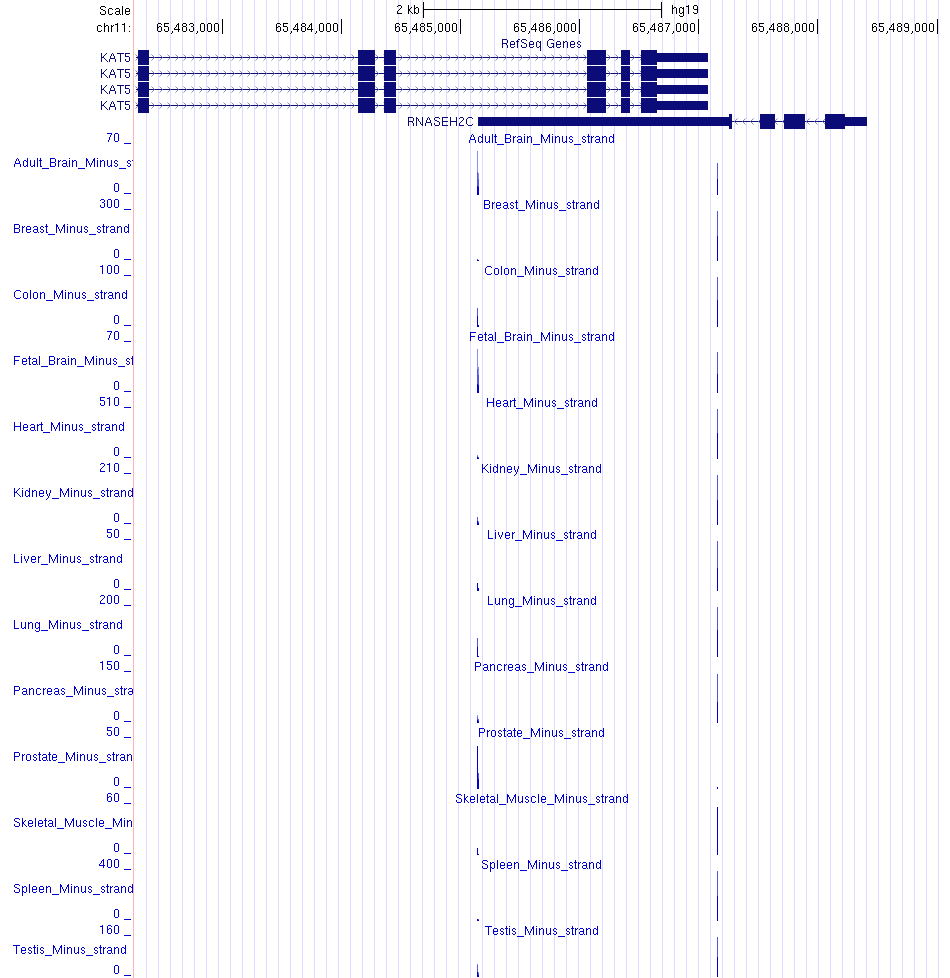


**Supplementary Figure S2. PA-seq tracks for *RNASEH2C* in 13 human tissues.** The wiggle files are downloaded from the published online material (1), and displayed on UCSC genome browser. Only minus strands were shown in UCSC genome browser.


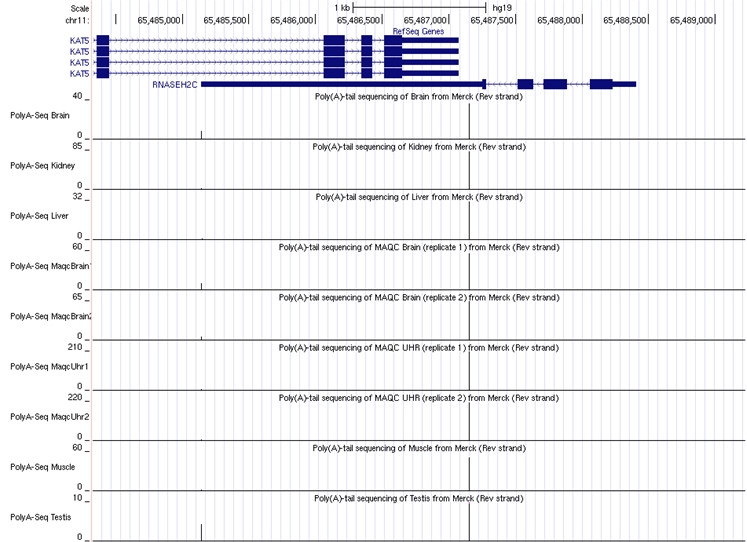


**Supplementary Figure S3. PolyA-Seq tracks for *RNASEH2C* in 9 human samples provided by UCSC genome browser.** Only reverse (or minus) strands were shown.


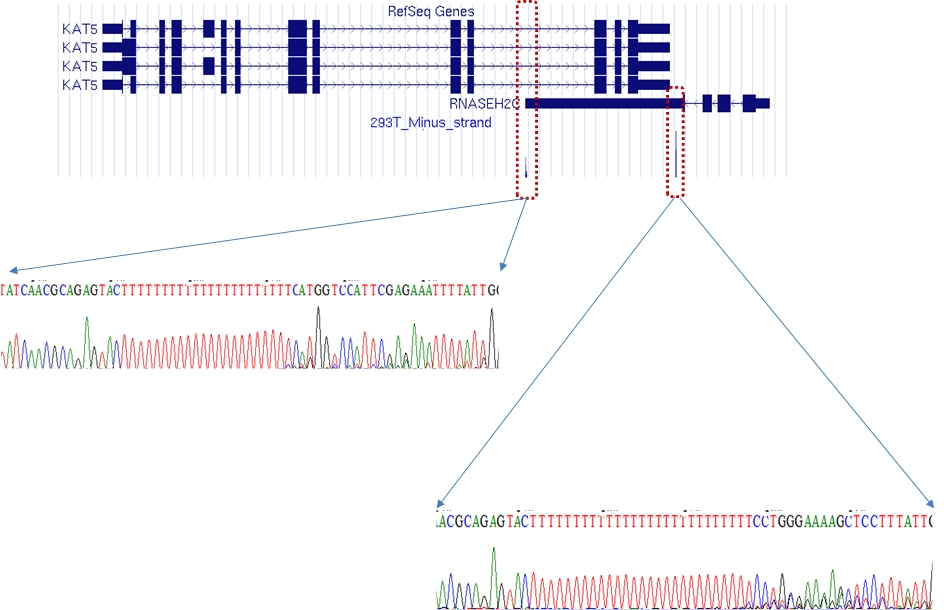


**Supplementary Figure S4. Sequences around the distal and proximal pA sites of human *RANSEH2C* by Sanger sequencing.** Human 293T cells was used for the sequencing validation.

**
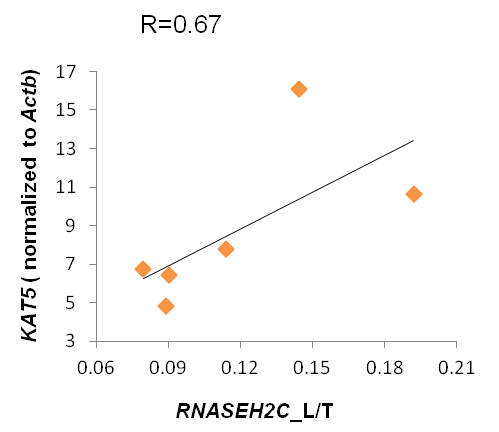
**

**Supplemental Figure S5. Antisense expression is associated with the usage frequency of distal pA site for *RNASEH2C* in human cells**. The relative expression of *KAT5* and the usage of distal pA site of *RNASEH2C* in six human cell lines were quantified by qRT-PCR.


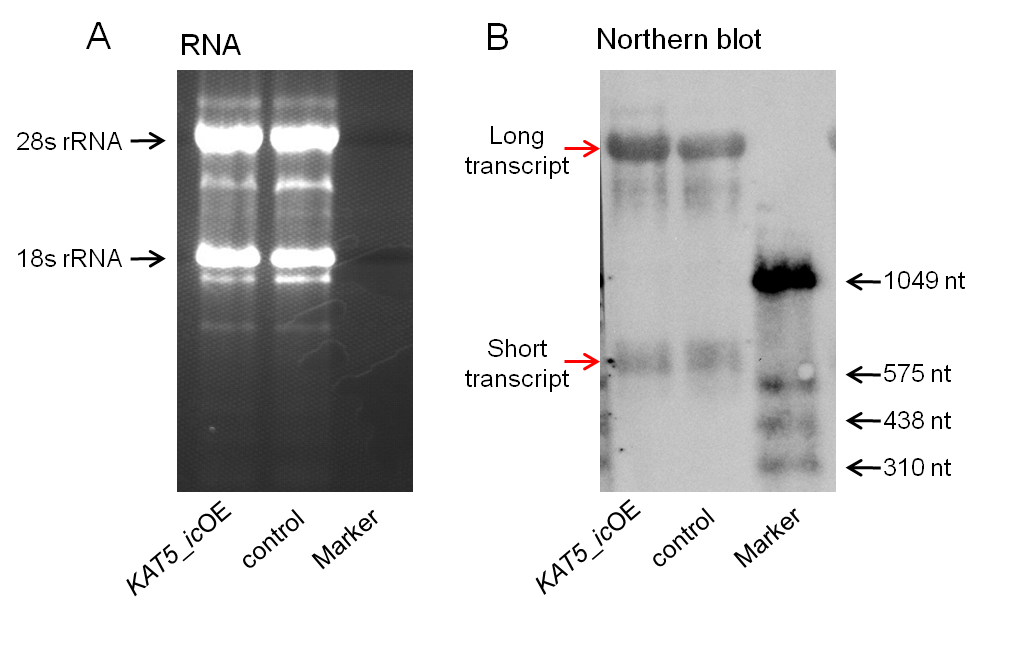


**Supplementary Figure S6. Northern blot for validation of the increased distal pA site usage in *in cis-*upregulated-*KAT5* cells. (**A) Agarose gel electrophoresis to confirm the quality of RNA. (B) Immunological detection of the long and short transcripts with DIG Northern Starter Kit. The long and short transcripts of *RNASEH2C* were marked by the red arrows.


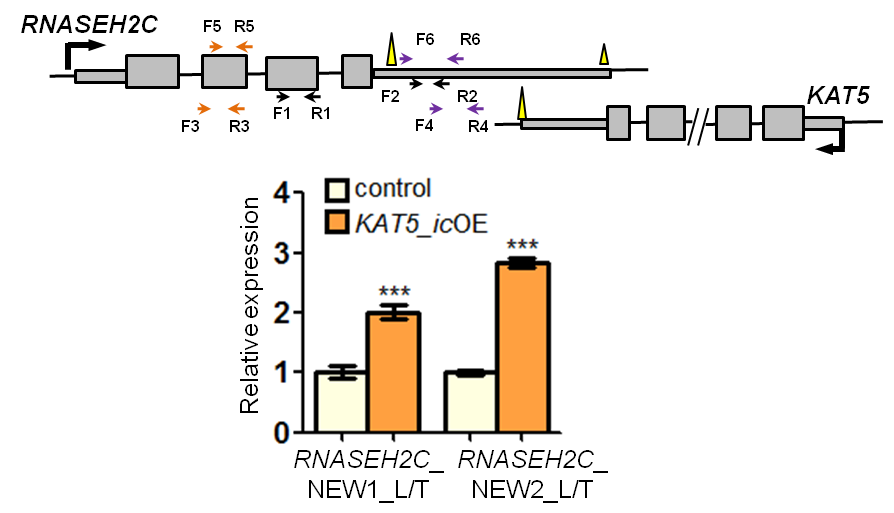


**Supplementary Figure S7. Detection of the distal pA site usage of *RNASEH2C* by evaluating the nascent RNA.** Use another four pairs of primers to detect the ratio of the long isoform expression level to that of the total of *RNASEH2C* in *KAT5*_*ic*OE cells and the control. NEW1: F3/R3 and F4/R4; NEW2: F5/R5 and F6/R6.


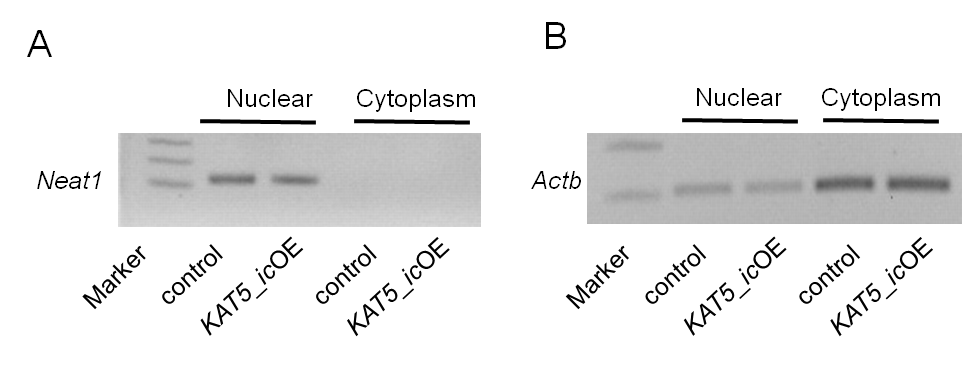


**Supplementary Figure S8.** Q**uality evaluation of nuclear and cytoplasmic fractionation.** (A-B). RT-PCR and agarose-gel detection for *Neat1* (the marker of nucleus) and *Actb* (the marker of cytoplasm), respectively.


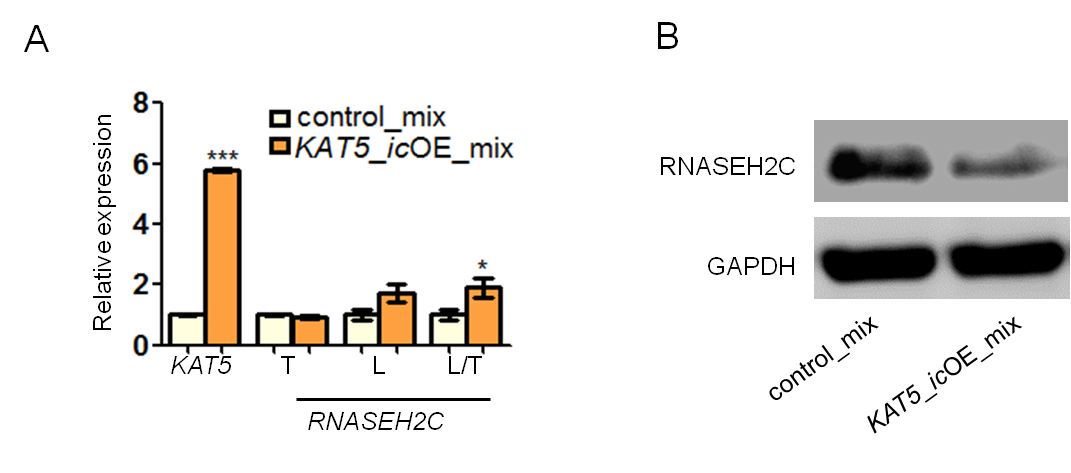


**Supplementary Figure S9. *In cis* upregulation of *KAT5* in mixed gene editing cells leads to higher usage of distal pA site and decreased protein abundance in *RNASEH2C*.** (A) The relative expression change of *KAT5* and the ratio of long isoform of *RNASEH2C* in cells with over-expression of *KAT5* *in cis* relative to control, quantified by qRT-PCR. (B) Western-blot for RNASEH2C upon over-expression *KAT5 in cis* and control. Gene editing mixed cells were used and GAPDH was used as the intern control in the assay. *, ** and *** represents p value (*t*-test) less than 0.05, 0.01 and 0.001compared to control, respectively.


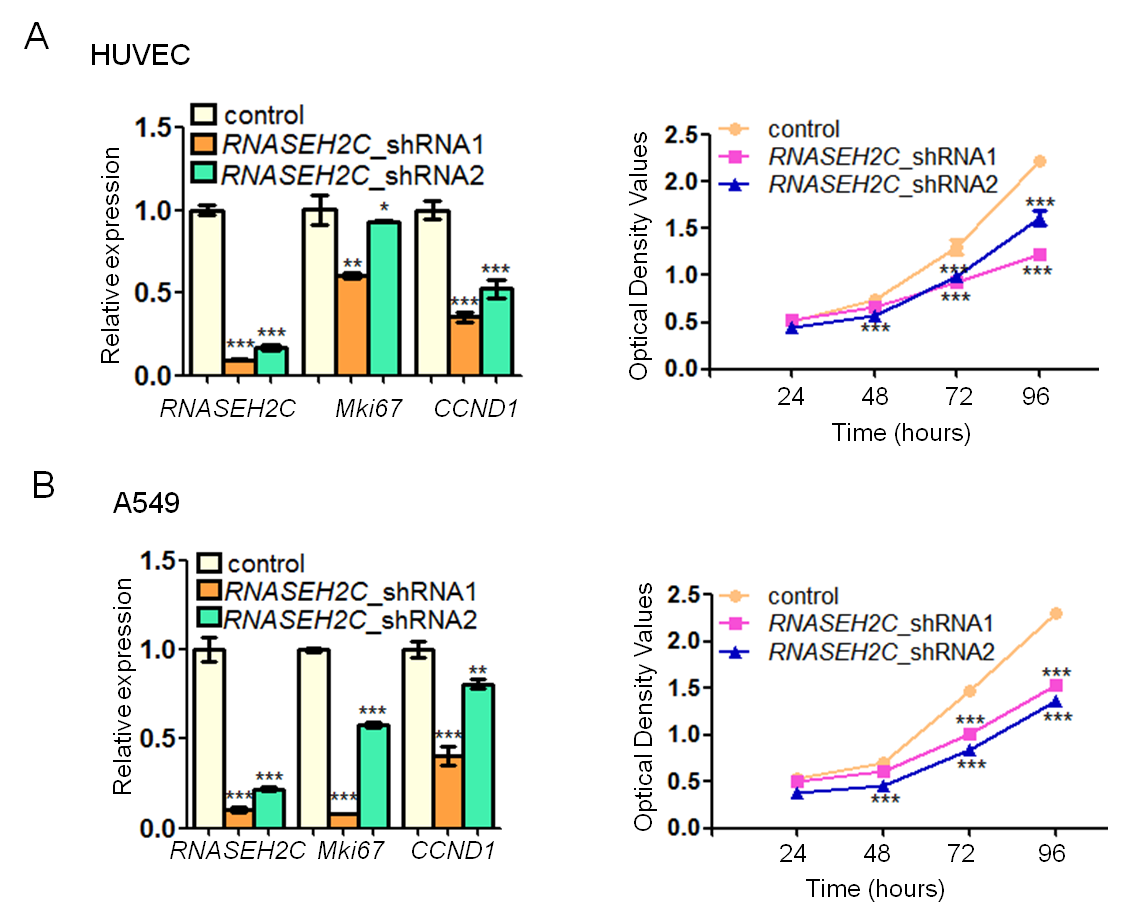


**Supplementary Figure S10. Knockdown of *RNASEH2C* in HUVEC and A549 cells leads to decreased cell proliferation.** (A-B) show the results acquired in HUVEC and A549 cells, respectively. The left panel was qRT-PCR for the relative expression level of *RNASEH2C*, *Mki67* and *CCND1*. The right panel was cell proliferation rate detection in *RNASEH2C*-knockdown cells compared to control cells assayed by CCK-8 kit. *, ** and *** represents p value (*t*-test) less than 0.05, 0.01 and 0.001compared to control, respectively.


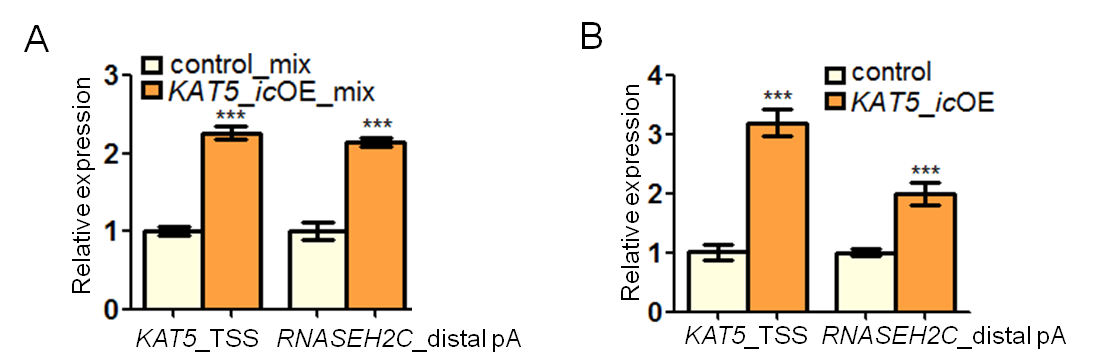


**Supplementary Figure S11. ChIP-qPCR validation for Pol II occupancy upon *KAT5 in cis* over-expression.** Results for mixed cells and single-clone-derived cells were represented in A and B, respectively. TSS denoted transcriptional start site of *KAT5*. *** means p value (*t*-test) less than 0.001 when compared *in cis* overexpression of KAT5 cells to the control.


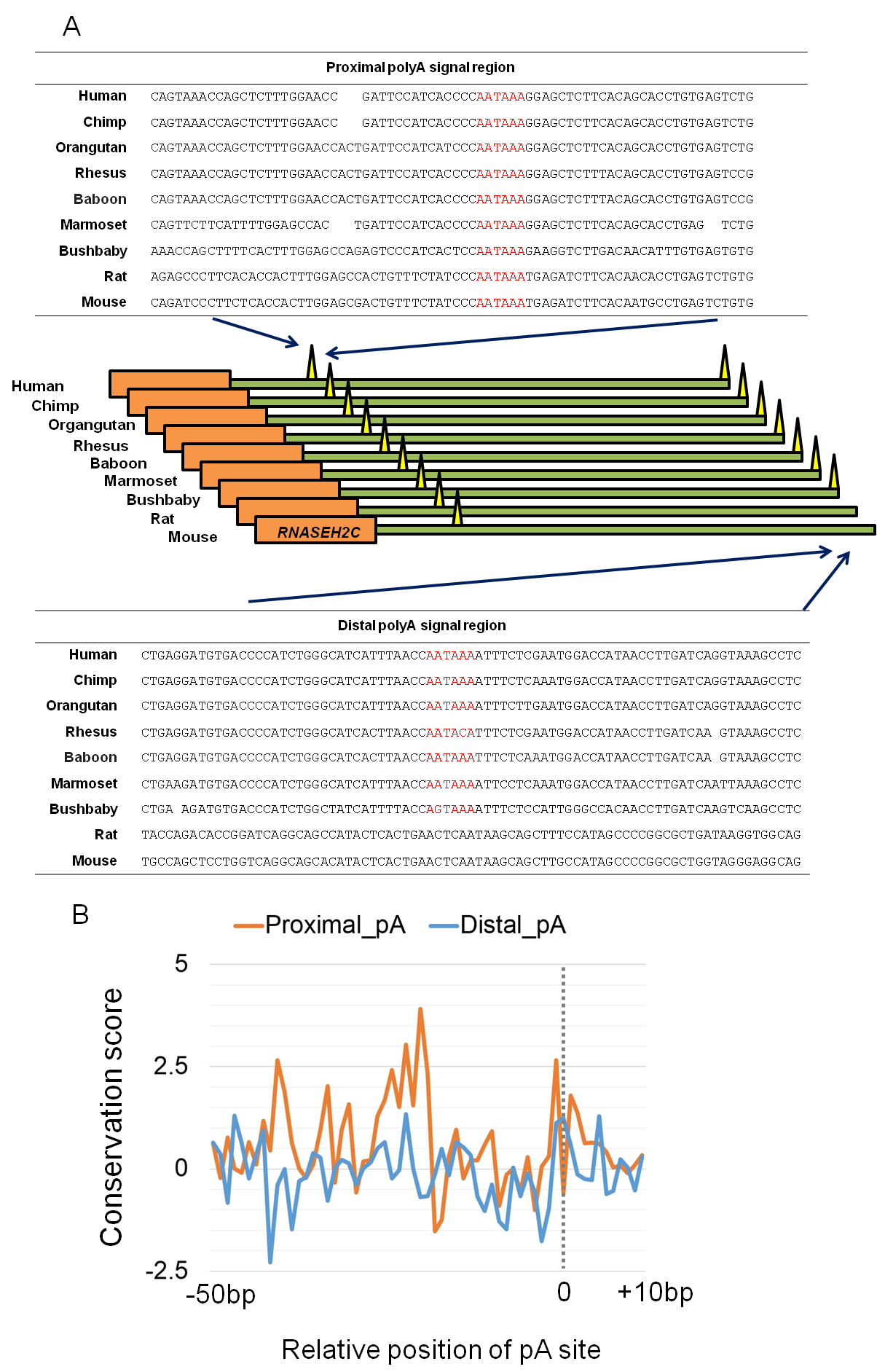


**Supplementary Figure S12. Conservation analysis of the proximal and distal pA site in *RNASEH2C*.** (A) The proximal and distal pA signal region in different species. (B) The conversation score for the sequences around the pA site.


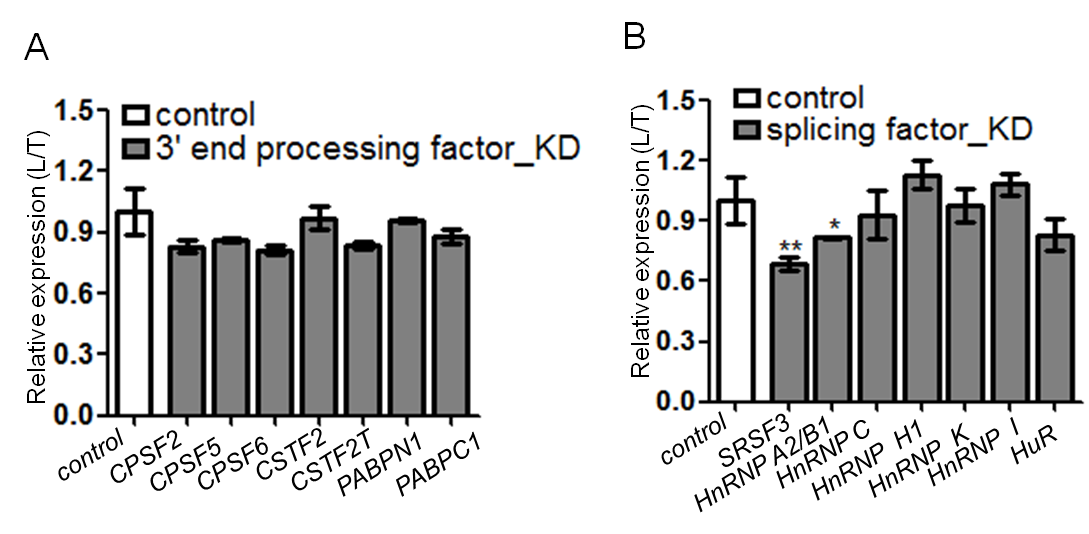


**Supplementary Figure S13. SRSF3 was involved in the regulation of pA site usage of *RNASEH2C*.** (A-B). qRT-PCR detection for the relative expression of the long isoform to total of *RNASEH2C* (L/T) after knockdown of several 3′ end processing factors (A) and splicing factors (B).


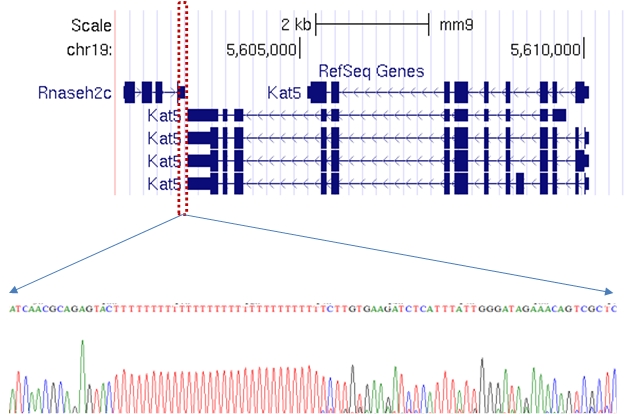


**Supplementary Figure S14. Sequence nearby the annotated pA sites of mouse *RANSEH2C* by Sanger sequencing.**


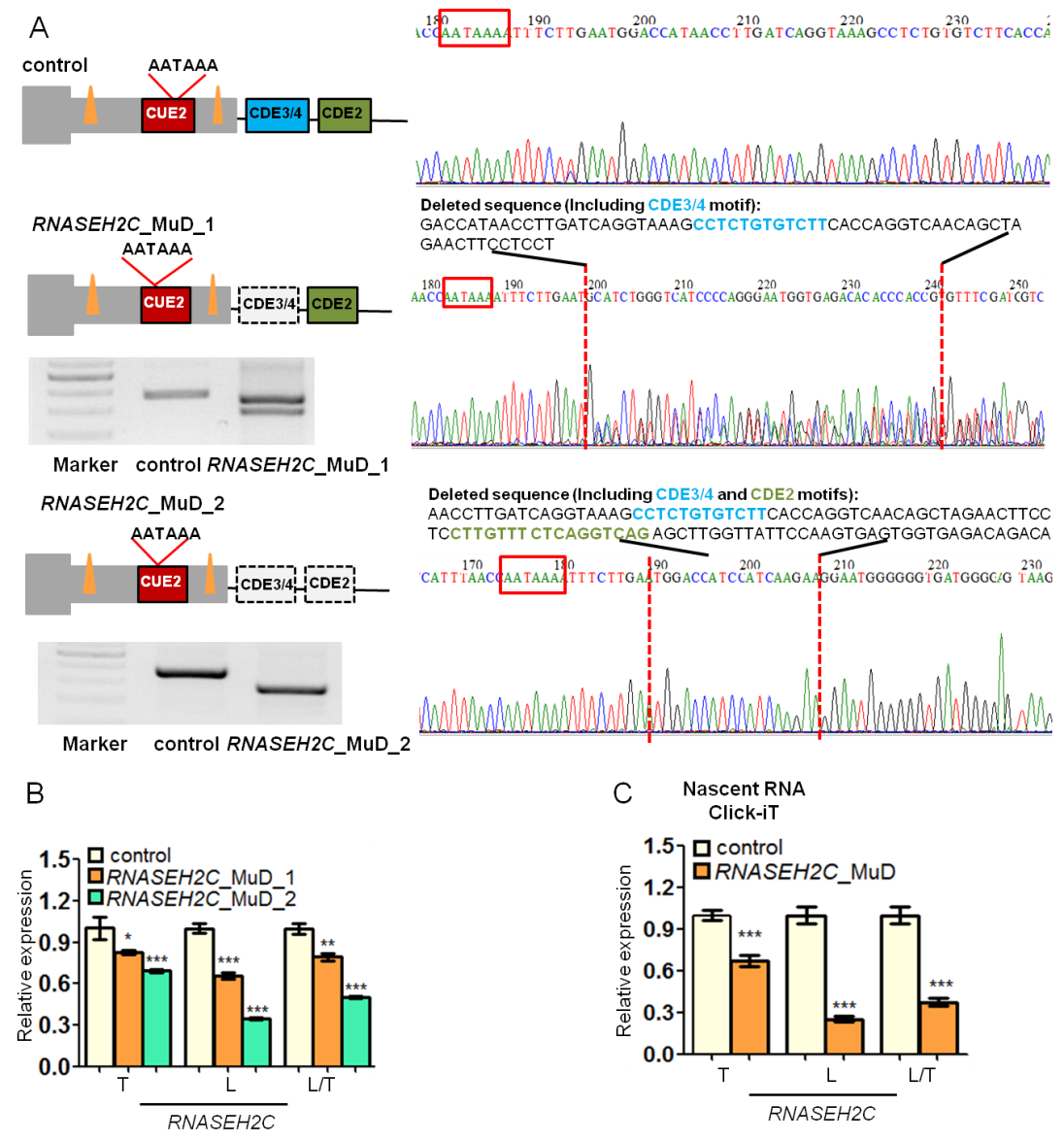


**Supplementary Figure S15. Depletion of downstream elements of distal pA site of *RNASEH2C* in human cells.** (A) Sanger sequencing confirms depletion of downstream elements of distal pA site of human *RNASEH2C*. The control that did not have any nucleotide mutation near the distal pA site was shown in the above panel. The left panel presented the *cis-acting* elements (CUE2, CDE2, CDE3 and CDE4) nearby the distal pA site of *RNASEH2C* predicted by PolyA_SVM server (2) and they were highlighted by colored boxes respectively. The orange arrows represented pA site. The right panel was the result of Sanger sequencing and CUE2 sequences were marked by a red box. The middle and bottom panel were the results of two mutant clones. The mutated *cis*-acting elements nearby the distal pA site were presented by gray dashed boxes in the left panel. In the right panel, the edited regions were underlined by red dotted lines and the deleted sequences were also shown. (B) The relative expression level of total isoform (T), long isoform (L) and ratio of long isoform to total isoform (L/T) of *RNASEH2C* were quantified by qRT-PCR compared *cis*-element mutated (MuD) cells to the control. (C) Nascent RNA analysis for the expression level of total, long isoform and the ratio of long isoform to the total (L/T) by qRT-PCR in *cis*-element mutated (MuD) cells and the control. *, ** and *** represents p value (*t*-test) less than 0.05, 0.01 and 0.001 compared to control, respectively.

**Supplementary Table 1. APA statistics in overlapped human genes.**

| **Category** | | **# of total genes** | **# of expressed genes** | **# of genes with single pA site** | **# of genes with multiple pA sites** |
| --- | --- | --- | --- | --- | --- |
| Genome | | 25371 | 14876 | 7706 | 7170 |
| Non-overlapped | | 19633 | 11405 | 6050 | 5355 |
| Overlapped | Complete overlap | 2069 | 1029 | 453 | 576 |
|  | Head-to-head | 1676 | 1045 | 522 | 523 |
|  | Tail-to-tail | 1993 | 1397 | 681 | 716 |

Head-to-head and tail-to-tail denoted sense and antisense genes overlapped in the 5′ and 3′ region, respectively. Complete overlap meaned one gene completely falled into the overlapped partner gene at the opposite strand.

**Supplementary Table 2. Primer sequences used in this study.**

| **Type** | **Gene name** | **Primer name** | **Primer sequence (5´ to 3´)** |
| --- | --- | --- | --- |
| qRT-PCR | *Actb* | Human_Actb-F | TAC GTT GCT ATC CAG GCT GT |
|  |  | Human_Actb-R | TCG TAG ATG GGC ACA GTG T |
|  | *GAPDH* | Mouse_GAPDH-F | AGGTCGGTGTGAACGGATTTG |
|  |  | Mouse_GAPDH-R | TGTAGACCATGTAGTTGAGGTCA |
|  | *RNASEH2C* | Human_RNASEH2C-F | ATT GGA GCC ACT GCC AAC T |
|  |  | Human_RNASEH2C-R | AAG GCT GGG CCA AGT TAA G |
|  |  | Human_RNASEH2C_Long-F | GTG AGT CTG GGC GCC TTA |
|  |  | Human_RNASEH2C_Long-R | AGC CAT GCA AAG TTC TTG GT |
|  |  | Mouse_RNASEH2C-F | AACTGAACTTCTCCGGGGAC |
|  |  | Mouse_RNASEH2C-R | CCCTATGCACTTTGGCATCT |
|  |  | Human_RNASEH2C-F3 | GTCTACGGGGAGAGGAGGTG |
|  |  | Human_RNASEH2C-R3 | CTCCTCTTGGTCGTCAGTCC |
|  |  | Human_RNASEH2C-F4 | GCAGCGATTCACGCACAG |
|  |  | Human_RNASEH2C-R4 | TGCTGGCTTAGGGGCTCT |
|  |  | Human_RNASEH2C-F5 | GGGACTCGAAGTGTCGTTTC |
|  |  | Human_RNASEH2C-R5 | CTCCTCCTCTTGGTCGTCAG |
|  |  | Human_RNASEH2C-F6 | CCGAGGACTGAGAACCAGAG |
|  |  | Human_RNASEH2C-R6 | TATATGCCAGAGCCATGCAA |
|  | *KAT5* | Human_KAT5-F | AGCCTGGTGTCTGATCGAAG |
|  |  | Human_KAT5-R | GGAGAAGTACCACGGCTTGA |
|  |  | Mouse_KAT5-F | AATCCCGGTCCAGATCACAC |
|  |  | Mouse_KAT5-R | CTGGGGTTGCTGGTGAAAC |
|  | *Mki67* | Human_Mki67-F | TCAAGACCCCAGTGAAGGAG |
|  |  | Human_Mki67-R | AGATGGCTGTTTTGCTGCAT |
|  | *CCND1* | Human_CCND1-F | CGTGGCCTCTAAGATGAAGG |
|  |  | Human_CCND1-R | CCACTTGAGCTTGTTCACCA |
|  | *SRSF3* | H_SRSF3-F | CGGCTTTGCTTTTGTTGAAT |
|  |  | H_SRSF3-R | TGGGCCACGATTTCTACTTC |
| ChIP-PCR and ChIP-qPCR | *KAT5* | H_KAT5CHIPPOLII-F | CGTTATGGGGTTTCCACCTA |
|  |  | H_KAT5CHIPPOLII-R | AGAGGGGGACCTTTGAGAGA |
|  | *RNASEH2C* | H_RNASEH2C_distal_pA-F | CTTCATTCTCCCCAGTCAGG |
|  |  | H_RNASEH2C_distal_pA-R | AGATGGGGTCACATCCTCAG |
| CRISPR/Cas9 | *KAT5* | H_KAT5_*ic*OE_gRNA1-F | TGGAGCGTTCGAACCCTTCTAGTTTTAGAG |
|  |  | H_KAT5_*ic*OE_gRNA1-R | CTAGCTCTAAAACTAGAAGGGTTCGAACGCTCCA |
|  |  | H_KAT5_*ic*OE_gRNA2-F | TGTCCGTCACGTGACGCCCACGTTTTAGAG |
|  |  | H_KAT5_*ic*OE_gRNA2-R | CTAGCTCTAAAACGTGGGCGTCACGTGACGGACA |
|  |  | H_KAT5_*ic*KD_gRNA1-F | TGGGCGTCTCCTTGTGGCGGC GTTTTAGAG |
|  |  | H_KAT5_*ic*KD_gRNA1-R | CTAGCTCTAAAACGCCGCCACAAGGAGACGCCCA |
|  |  | M_KAT5_*ic*OE_gRNA1-F | TGCAAAAAGCCGGCCCGTTTGGTTTTAGAG |
|  |  | M_KAT5_*ic*OE_gRNA1-R | CTAGCTCTAAAACCAAACGGGCCGGCTTTTTGCA |
|  |  | M_KAT5_*ic*OE_gRNA2-F | TGTCCGGAGCTGAGTGCGCTATGTTTTAGAG |
|  |  | M_KAT5_*ic*OE_gRNA2-R | CTAGCTCTAAAACATAGCGCACTCAGCTCCGGACA |
|  |  | U6-F-XbaI | AGTCTAGAGTCTTGACAGCAGACCTCGT |
|  |  | gRNA-R-XhoI | GACTCTCGAGCGATGCTAGC |
|  |  | puro-CMV-F | TCCGACTTCAACTGTAGGGTTGCTTAGGGTTAGGCGTTTT |
|  |  | CMV-R | AGCCAGTAAGCAGTGGGTTC |
|  |  | PURO-F | TGTGTCAGTTAGGGTGTGGA |
|  |  | PURO-R | ACCCTACAGTTGAAGTCGGA |
|  |  | H_KAT5_Up-F | AGAACCTGTACACCCTCCAC |
|  |  | H_KAT5_Up-R | TCCACACCCTAACTGACACAGCAAGAGGACTCAGGGAAGA |
|  |  | H_KAT5-CMV & Down-F | GAACCCACTGCTTACTGGCTCGTTATGGGGTTTCCACCTA |
|  |  | H_KAT5-puro & Down-F | TCCGACTTCAACTGTAGGGTCGTTATGGGGTTTCCACCTA |
|  |  | H_KAT5-Down-R | GGATGGCTCAGGACAGAATG |
|  |  | M_KAT5_Up-F | TTGAGGAGGGAGAACACACC |
|  |  | M_KAT5_Up-R | TCCACACCCTAACTGACACAGACGTTGTAAACCTGAGGCC |
|  |  | H_KAT5-CMV & Down-F | GAACCCACTGCTTACTGGCTCAGTGGAGGGAGGGAAGATG |
|  |  | H_KAT5-puro & Down-F | TCCGACTTCAACTGTAGGGTCAGTGGAGGGAGGGAAGATG |
|  |  | M_KAT5_Down-R | ACTCACCACCTCTCTTTCGG |
|  |  | H_KAT5_donor-F | CATCCACAGGTACACCTCCA |
|  |  | H_KAT5_donor-R | CTCTGGGAGACGTCACTTCC |
|  |  | H_KAT5_*ic*KD_T7 EI-F | TTACAGGGAGCTGAGATCGC |
|  |  | H_KAT5_*ic*KD_T7 EI-R | CTCACGAAGCCCCTGTAGAG |
|  |  | M_KAT5_donor-F | CATCCACAGGTACACCTCCA |
|  |  | M_KAT5_donor-R | ACTGATGTCCTTCACGCTCA |
|  | *RNASEH2C* | H_RNASEH2C_MuD-gRNA1-F | CGCTCTTCGCCGGCTTTACCTGATCAAGGTTAGTTTTAGAGCTAGAAATAGCAA |
|  |  | H_RNASEH2C_MuD-gRNA2-R | CGCTCTTCTAACTCTGAGGATGTGACCCCATCCGGTGTTTCGTCCTTTCCAC |
|  |  | H_RNASEH2C_MuD_T7 EI-F | CTTCATTCTCCCCAGTCAGG |
|  |  | H_RNASEH2C_MuD_T7 EI-R | CAGCCTCTTACTCCCCATCA |
| shRNA | *RNASEH2C* | H_RNASEH2CshRNA1-F | CCGGGACTTCGACCGCTTCATTGGACTCGAGTCCAATGAAGCGGTCGAAGTCTTTTTTG |
|  |  | H_RNASEH2CshRNA1-R | AATTCAAAAAAGACTTCGACCGCTTCATTGGACTCGAGTCCAATGAAGCGGTCGAAGTC |
|  |  | H_RNASEH2CshRNA2-F | CCGGGTGACAGAAGAGAAGAAGGTGCTCGAGCACCTTCTTCTCTTCTGTCACTTTTTTG |
|  |  | H_RNASEH2CshRNA2-R | AATTCAAAAAACAATCACCAGCCCTCTGTACACTCGAGTGTACAGAGGGCTGGTGATTG |
| Dual luciferase assay | *RNASEH2C* | H_RNASEH2Cshort&long3'UTR-F | CTCGAGCACAGGTGCCCGAGGACT |
|  |  | H_RNASEH2Cshort3'UTR-R | GCGGCCGCTGCTGTGAAGAGCTCCTTTATT |
|  |  | H_RNASEH2Clong3'UTR-R | GCGGCCGCTTATGGTCCATTCGAGAAATTTTA |
|  |  | H_RNASEH2Cmutantlong3'UTR-F | GGAGCTCTTCACAGCACCT |
|  |  | H_RNASEH2Cmutantlong3'UTR-R | CAATTGGGGGTGATGGAATC |
| *KAT5* Overexpression | *KAT5* | H_KAT5_OE-F | CGGAATTCGCCACCAGTGGAGGGAGGGAAGATG |
|  |  | H_KAT5_OE-R | CGCGGATCCCCACTTCCCCCTCTTGCT |
| *KAT5* knockdown | *KAT5* | H_KAT5_siRNA | rArGrArArUrCrArArCrGrGrArArGrArCrUrArCrArArUrGTG |
| Northern blot | *RNASEH2C* | H_RNASEH2C-F | GATCACTAATACGACTCACTATAGGGGGCACCTGTGCGTGAATC |
|  |  | H_RNASEH2C-R | GGGACTCGAAGTGTCGTTTC |

REFERENCES

1. Ni T, Yang Y, Hafez D, Yang W, Kiesewetter K, Wakabayashi Y, et al. Distinct polyadenylation landscapes of diverse human tissues revealed by a modified PA-seq strategy. BMC genomics. 2013;14:615.

2. Hu J, Lutz CS, Wilusz J, Tian B. Bioinformatic identification of candidate cis-regulatory elements involved in human mRNA polyadenylation. Rna. 2005;11(10):1485-93.
